# Acceleration of brain aging after small-volume infarcts

**DOI:** 10.3389/fnagi.2024.1409166

**Published:** 2024-09-19

**Authors:** Ying-Ju Peng, Chen-Yuan Kuo, Sheng-Wei Chang, Ching-Po Lin, Yuan-Hsiung Tsai

**Affiliations:** ^1^Department of Diagnostic Radiology, Chang Gung Memorial Hospital, Chiayi, Taiwan; ^2^Department of Diagnostic Radiology, Chang Gung University, Taoyuan, Taiwan; ^3^Department of Neurology, Neurological Institute, Taipei Veterans General Hospital, Taipei, Taiwan; ^4^Institute of Neuroscience, National Yang Ming Chiao Tung University, Taipei, Taiwan; ^5^Department of Education and Research, Taipei City Hospital, Taipei, Taiwan

**Keywords:** aging, stroke, retrospective studies, brain, magnetic resonance imaging, machine learning, neuroimaging, infarction

## Abstract

**Introduction:**

Previous studies have shown that stroke patients exhibit greater neuroimaging-derived biological “brain age” than control subjects. This difference, known as the brain age gap (BAG), is calculated by comparing the chronological age with predicted brain age and is used as an indicator of brain health and aging. However, whether stroke accelerates the process of brain aging in patients with small-volume infarcts has not been established. By utilizing longitudinal data, we aimed to investigate whether small-volume infarctions can significantly increase the BAG, indicating accelerated brain aging.

**Methods:**

A total of 123 stroke patients presenting with small-volume infarcts were included in this retrospective study. The brain age model was trained via established protocols within the field of machine learning and the structural features of the brain from our previous study. We used *t*-tests and regression analyses to assess longitudinal brain age changes after stroke and the associations between brain age, acute stroke severity, and poststroke outcome factors.

**Results:**

Significant brain aging occurred between the initial and 6-month follow-ups, with a mean increase in brain age of 1.04 years (*t* = 3.066, *p* < 0.05). Patients under 50 years of age experienced less aging after stroke than those over 50 years of age (*p* = 0.245). Additionally, patients with a National Institute of Health Stroke Scale score >3 at admission presented more pronounced adverse effects on brain aging, even after adjusting for confounders such as chronological age, sex, and total intracranial volume (*F*_1,117_ = 7.339, *p* = 0.008, *η*^2^ = 0.059). There were significant differences in the proportional brain age difference at 6 months among the different functional outcome groups defined by the Barthel Index (*F*_2,118_ = 4.637, *p* = 0.012, *η*^2^ = 0.073).

**Conclusion:**

Stroke accelerates the brain aging process, even in patients with relatively small-volume infarcts. This phenomenon is particularly accentuated in elderly patients, and both stroke severity and poststroke functional outcomes are closely associated with accelerated brain aging. Further studies are needed to explore the mechanisms underlying the accelerated brain aging observed in stroke patients, with a particular focus on the structural alterations and plasticity of the brain following minor strokes.

## Introduction

Stroke is the second leading cause of death and a major cause of disability worldwide (Katan and Luft, 2018). Stroke is one of the most common causes of acquired cognitive disabilities during adulthood, and its prevalence is projected to increase in the coming decades due to the aging population (Feigin et al., 2016). Previous studies have shown that stroke patients experience accelerated brain aging before a stroke occurs, and a stroke event further accelerates the brain aging process (Egorova et al., 2019). Brain aging is one of the most crucial biological processes affecting the physiological balance between health and disease. Age-associated dysfunction of the brain leads to severe health problems in the current aging society (Danka Mohammed et al., 2017). During acute ischemic stroke, for each hour in which there is no treatment, the brain loses as many neurons as it does in almost 3.6 years of normal aging. This means that 1 h of ischemic brain damage is equivalent to 3.6 years of normal brain aging (Saver, 2006). The observed brain changes not only suggest potential vascular-related brain damage but also indicate accelerated alterations in the underlying biological age of the brain (Werden et al., 2017).

Advanced neuroimaging techniques, which utilize MRI within a machine learning framework for estimating biological brain age, are currently widely employed (Cole and Franke, 2017; Franke and Gaser, 2019). The differences observed between chronological age and predicted brain age, referred to as the brain age gap (BAG), serve as a reflection of brain health and aging. Studies have longitudinally examined the BAG between stroke survivors and control participants, matched in chronological age, and suggest that stroke may be an ultimate manifestation of gradual cerebrovascular burden accumulation and brain degeneration (Egorova et al., 2019). An investigation explored the association between the BAG and poststroke neurocognitive disorders over time and revealed that a younger-appearing brain is associated with a lower risk of poststroke neurocognitive disorder (NCD) (Aamodt et al., 2023). The main effects of the BAG on cognitive performance and the associations between the BAG and task improvement have also been tested, suggesting that longitudinal brain age prediction based on automated brain morphometry is feasible and reliable in stroke patients. However, no significant associations between brain age and either performance and/or response to cognitive training have been reported (Richard et al., 2020). Furthermore, no study has addressed the longitudinal observation of brain age in small-volume infarct patients and its correlation with poststroke disease evolution or functional outcomes.

In this study, we conducted a descriptive and retrospective analysis of stroke patients with small-volume infarcts to investigate differences between brain age and chronological age at both the initial assessment and the 6-month follow-up after stroke. We also explored potential correlations between brain age and poststroke functional outcomes. Our hypotheses are as follows: first, we expect stroke patients with small-volume infarcts to show a greater increase in predicted brain age in the 6-month follow-up analysis than in the initial assessment within 1 month after stroke onset. Second, we anticipate that patients with more severe strokes will experience more pronounced accelerated brain aging over time than those with less severe strokes. Finally, we expect that stroke patients who demonstrate improved basic activities of daily living and better clinical outcomes experience a relatively delayed brain aging process, which is indicative of a greater cognitive or brain reserve.

## Materials and methods

### Participants

A total of 123 patients with small-volume infarcts were enrolled in this study. Small-volume infarcts were defined as baseline diffusion-weighted imaging (DWI) restriction lesions ≤5 mL (Jackson and Sudlow, 2005; De Silva et al., 2013). Furthermore, T1-weighted MRI data from 2,675 healthy participants (aged 18–92 years, 1,247 men and 1,428 women) were collected as a training dataset at five different sites to construct a structural covariance network (SCN)-based brain age prediction model (Huang et al., 2020; Liu et al., 2020; Chou et al., 2021; Lee et al., 2018; Wei et al., 2021; Kuo et al., 2023). All participants had no prior history of head trauma, brain lesions, significant medical conditions, or neurological/neuropsychiatric disorders.

### Data collection and clinical characteristics

The data for this study were retrospectively collected from the medical records of the patients. The collected information included age, sex, level of education, and various cardiovascular risk factors, such as hypertension, smoking status, diabetes mellitus, and hyperlipidemia. Additionally, the prestroke modified Rankin scale (mRS) score was recorded to assess patients’ dependence on performing daily activities before the stroke event. Stroke details, including the Trial of Org 10,172 in Acute Stroke Treatment (TOAST) classification and the location of the stroke, were also documented. Acute stroke severity was measured via the National Institutes of Health Stroke Scale (NIHSS) score at the emergency department and upon admission. The administration of intravenous tissue-type plasminogen activator treatment or endovascular thrombectomy was also recorded. Other relevant outcome factors, such as the length of hospital stay, mortality, the occurrence of stroke in evolution, and postacute care (PAC) services provided to patients after an acute stroke during their hospitalization, were also recorded. The functional outcome assessments included the poststroke mRS score and Barthel Index score at 3–6 months after stroke.

### MRI acquisition

MRI scans were conducted at two time points: baseline (between 2 and 7 days after the onset of acute stroke) and at a 6-month follow-up. The assessments were performed using a 1.5-T Philips Gyroscan Intera scanner (Philips Medical Systems). The MRI protocol included 3D-T1 weighted, axial and sagittal T2, 3D-fluid attenuated inversion recovery (FLAIR), diffusion-weighted imaging (DWI), and 3D time-of-flight (TOF) magnetic resonance angiography.

### Brain imaging processing and analysis

To evaluate conventional MRI-based markers associated with small vessel disease (SVD), we quantified the volume of white matter hyperintensities (WMHs). The LesionBrain application, available on the VolBrain website,^1^ facilitated the automatic segmentation and volume measurement of WMHs on T1- and T2-FLAIR-weighted images (Romero et al., 2018).

Stroke volume was determined by identifying a hyperintense signal on DWI along with a corresponding hypointense signal on the apparent diffusion coefficient (ADC) map. Lesions were manually traced on the DWI image. This tracing process was conducted by a well-experienced neuroradiologist via RadiAnt DICOM Viewer (64-bit) software. Lesion volumes were then computed in cubic centimeters (cm^3^).

Intracranial atherosclerotic disease (ICAD) was defined as the presence of diseases affecting the intracranial internal carotid, middle cerebral, or basilar arteries. ICAD was further classified based on the degree of narrowing of the luminal diameter, with categories including normal, <50% stenosis, ≥50% stenosis, or occlusion (Uehara et al., 1996). In this study, the presence of ICAD was defined as moderate-to-severe stenosis, which corresponds to 50 to 99% luminal narrowing in the intracranial artery. The identification and verification of ICAD were performed visually on the TOF angiography data of each subject. This process was carried out by a neuroradiologist via RadiAnt DICOM Viewer (64-bit) software.

### Brain age prediction model construction

Individual maps of gray matter volume and density were created from T1-weighted scans via a voxel-based morphometry analysis pipeline in Statistical Parametric Mapping 12 within MATLAB (version R2021a; MathWorks, Natick, MA). The T1-weighted images in the training dataset underwent a six-step process (Lee et al., 2022; Kuo et al., 2021), including correction for bias-field homogeneities; segmentation into gray matter (GM), white matter (WM), and cerebrospinal fluid (CSF); creation of a study-specific tissue template; spatial normalization to the standard Montreal Neurological Institute (MNI) space via the Diffeomorphic Anatomical Registration Through Exponentiated Lie Algebra (DARTEL) approach (Ashburner, 2007); modulation of tissue densities for volumetric preservation; spatial normalization to 1.5-mm isotropic voxels; and spatial smoothing via a 6 mm full width at half maximum (FWHM) Gaussian kernel. Maps of modulated gray matter volume (GMV) and unmodulated gray matter density (GMD) were subsequently estimated in the training dataset for further feature extraction.

For data reduction and feature extraction, our previous studies comprehensively outlined the framework for spatial independent component analysis (sICA)-based feature extraction (Kuo et al., 2021; Kuo et al., 2020). In essence, the estimated GMV and GMD were consolidated into two distinct 4D datasets. The use of sICA on the training dataset involved applying the Multivariate Exploratory Linear Optimization Decomposition into Independent Components (MELODIC; FSL v5.0.9)^2^ tool, which decomposed these concatenated 4D GMVs and GMDs into 400 spatially distinct components each (Kuo et al., 2021). Then, spatial regression analysis was applied to calculate the integrity score for each independent component in the training dataset and was applied to the clinical dataset. These 800 feature values for each participant, comprising 400 for GMV and 400 for GMD, function as input features for the subsequent development of machine learning-based brain age models in the training dataset (Uehara et al., 1996; Ashburner, 2007).

Our proposed brain age prediction model used the radial basis function (RBF) kernel-based support vector regression (SVR) algorithm for training. The hyperparameters considered included C and gamma, spanning specific values from 0.001- to 1,000, while other parameters were maintained at default values. This optimization process involved a fivefold cross-validation scheme to fine-tune the hyperparameters on the training dataset. The final brain age prediction model subsequently underwent retraining with the selected hyperparameters and was then applied to predict brain age in the clinical dataset. Please refer to our previous study for a detailed overview of the training architecture (Kuo et al., 2023).

As commonly performed in studies comparing predicted brain age, the individual BAG was computed by subtracting the predicted brain age from the chronological age of each individual in the clinical dataset at the onset of stroke and at the 6-month follow-up. Furthermore, the difference between the initial and 6-month BAG (dBAG) was calculated to assess longitudinal changes (DeLong et al., 2022). The relative disparity between the estimated brain age and the patient’s chronological brain age was quantified via the following formula: (estimated brain age-chronological age)/chronological age. This parameter is referred to as the proportional brain age difference (PBAD). Positive PBAD values indicate advanced brain aging, whereas negative values suggest delayed brain aging (Busby et al., 2022).

### Statistical analysis

Statistical analyses were conducted via MedCalc statistical software and the Statistical Package for the Social Sciences version 25 (SPSS; IBM Corp., Armonk, NY, United States). Means, standard deviations (SDs), and additional medians with interquartile values were computed for relevant data. First, a paired *t*-test was used to assess whether stroke accelerated brain aging in the longitudinal analysis, by comparing the BAGs at the initial assessment and the 6-month follow-up. We subsequently investigated the hypothesis that the severity of acute stroke may contribute to accelerated brain aging. We defined those with NIHSS scores above the median at admission (median = 3, interquartile range: 2–5) as the higher group and those with scores of 3 or less as the lower group. To identify changes in dBAG between the higher and lower NIHSS score groups, we employed analysis of variance and analysis of covariance (ANCOVA), adjusting for chronological age and the square of chronological age, sex, and total intracranial volume (TIV) as nuisance variables.

Moreover, among the stroke patients, we performed univariate analysis with PBAD at 6 months as the dependent variable with various outcome factors, including stroke evolution, poststroke mRS score, and the Barthel index score, as the independent variables. Sex and TIV were used as nuisance variables. The objective of this study was to investigate whether patients who demonstrated better basic activities of daily living and improved clinical outcomes after stroke exhibited a relatively delayed brain aging process, which could be indicative of a greater cognitive or brain reserve.

## Results

### Study population

The population demographics and clinical characteristics of the patients are summarized in Table 1. Among the participants, 64.2% were women, and the mean chronological age was 65.53 years (SD = 11.31). The NIHSS scores at the emergency department and at admission were 3.41 (SD = 2.52) and 3.69 points (SD = 3.09), respectively, indicating that most of the strokes were mild. The median prestroke mRS score was 0 points (IQR: 0–0), suggesting that the patients had minimal to no disability before the stroke event.

**Table 1:** Demographics and sample characteristics of the stroke patients.

	Mean (SD)
Total *N* (% females)	123 (64.2%)
Chronological age (years)	65.53 (11.31)
Hypertension	92 (74.8%)
Smoking	51 (41.5%)
Diabetes mellitus	43 (35%)
Hyperlipidemia	40 (32.5%)
ivTPA	0 (0%)
ICAD on MRA	11 (8.9%)
NIHSS score at ER department (points)	3.41 (2.52)
NIHSS score at admission (points)	3.69 (3.09)
Prestroke mRS score (points)	0 (0–0)*
WMH Vol (V%)	0.39 (0.66)
WMH Vol (cm^3^)	5.25 (9.51)
Stroke volume (cm^3^)	5.09 (5.71)
Stroke location	
Left	47 (38.2%)
Right	50 (40.7%)
Brain stem/cerebellum	20 (16.3%)
Bilateral	6 (4.9%)
TOAST classification	
Large artery atherosclerosis	0 (0%)
Cardioembolism	2 (1.6%)
Small-vessel occlusion	91 (74%)
Other/undetermined	30 (24.4%)
Length of stay (days)	10.79 (9.43)
Death	0 (0%)
Stroke in evolution	10 (8.1%)
Postacute care (PAC)	12 (9.8%)
Barthel index score (points)	74.79 (24.94)
Poststroke mRS score (points)	2.67 (1.42)

*Presented as the median (IQR).

At baseline, the comorbidities and risk factors observed in the study population included hypertension (*n* = 92, 74.8%), smoking (*n* = 51, 41.5%), and diabetes mellitus (*n* = 43, 35%). None of the patients received intravenous tissue-type plasminogen activator treatment or endovascular thrombectomy. ICAD detected through MRA was present in 11 patients (8.9%). The mean volume of the WMHs was 5.25 cm^3^ (SD = 9.51), which was 0.39% (SD = 0.66) relative to the total intracranial volume. The mean stroke lesion volume was 5.09 cm^3^ (SD = 5.71). Among the study participants, right-hemisphere lesions were the most common (50 cases, 40.7%), followed by left-hemisphere lesions (47 cases, 38.2%). Additionally, six patients (4.9%) presented with acute bilateral infarctions. With respect to the distribution of stroke locations, subcortical lesions were the most prevalent, occurring in 65 cases (52.8%), followed by cortical lesions in 37 cases (30.1%), brainstem lesions in 19 cases (15.4%), and cerebellar lesions in two cases (1.6%). According to the TOAST classification, the most prevalent subtype of stroke was small-vessel occlusion, accounting for 91 cases (74%). Other or undetermined causes were observed in 30 patients (24.4%).

In terms of the outcome factors, the mean hospital stay length was 10.79 days (SD = 9.43). There were no reported deaths (*n* = 0, 0%) during the study period. Stroke-in-evolution occurred in 10 patients (8.1%), and 12 patients (9.8%) received postacute care after stroke (PAC). The mean Barthel Index score was 74.79 points (SD = 24.94), and the mean poststroke mRS score was 2.67 points (SD = 1.42).

### Longitudinal analysis of brain age in stroke patients between the initial assessment and the 6-month follow-up

**Figure 1:**
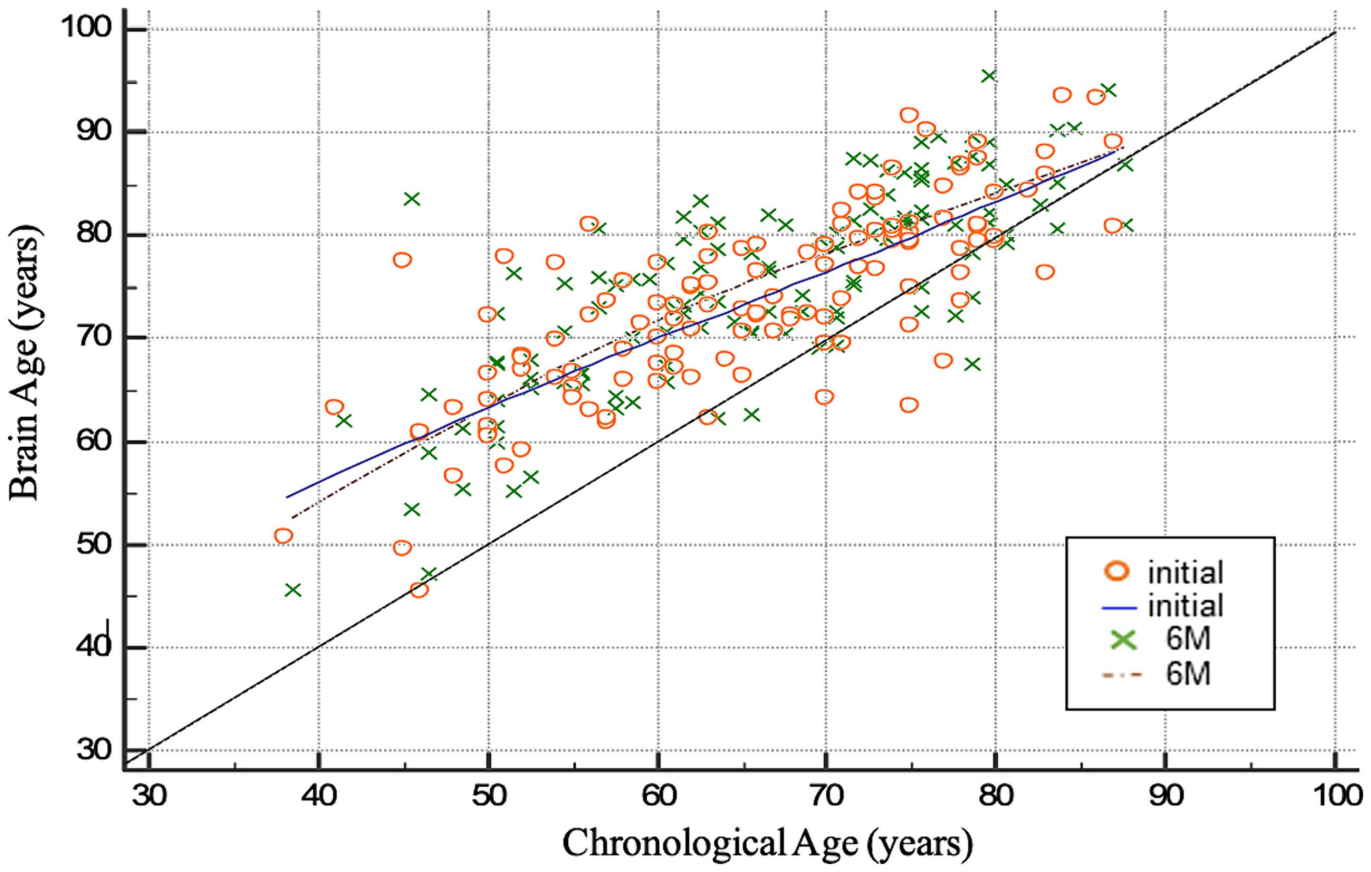
Scatter plots of chronological age by brain age. Dots representing the patients are colored orange (trend line in blue) at the initial follow-up visit and green at the 6 M (6-month) follow-up visit (trend line in brown). Note the tendency for greater brain age at the 6-month follow-up in patients above 50 years of chronological age (brown dotted line crossing the blue line).

The demographic characteristics of stroke patients related to factors associated with brain aging are summarized in Table 2. The mean initial brain age was 73.71 years (SD = 9.17), and the mean brain age at the 6-month follow-up was 75.24 years (SD = 9.65). The mean initial BAG was 8.18 years (SD = 7.12), and the mean BAG at 6 months was 9.22 years (SD = 7.30). The mean dBAG was 1.04 years (SD = 3.75). The mean initial proportional brain age difference (PBAD) was 0.14 (SD = 0.14), whereas the mean PBAD at 6 months was 0.15 (SD = 0.14). The correlations between brain age and chronological age, based on the data obtained at the initial assessment and the 6-month follow-up, are presented in Figure 1. Compared to the older group (≥ 50 years), with a mean dBAG of 1.121 ± 3.76, the younger group (< 50 years) had a smaller mean dBAG of −0.036 ± 3.57. However, this difference was not statistically significant (*F*_1,116_ = 1.363, *p* = 0.245, *η*^2^ = 0.012).

**Table 2:** Demographics of stroke patients regarding brain age factors (*n* = 123).

	Mean (SD)
Brain age initial (years)	73.71 (9.17)
BAG initial (years)	8.18 (7.12)
Brain age 6 months (years)	75.24 (9.65)
BAG 6 months (years)	9.22 (7.30)
dBAG (years)	1.04 (3.75)
PBAD initial	0.14 (0.14)
PBAD 6 months	0.15 (0.14)
GMV initial (cm^3^)	547.62 (63.85)
TIV initial (cm^3^)	1366.23 (124.18)
GMV 6 months (cm^3^)	543.02 (63.98)
TIV 6 months (cm^3^)	1371.86 (121.96)

**Figure 2:**
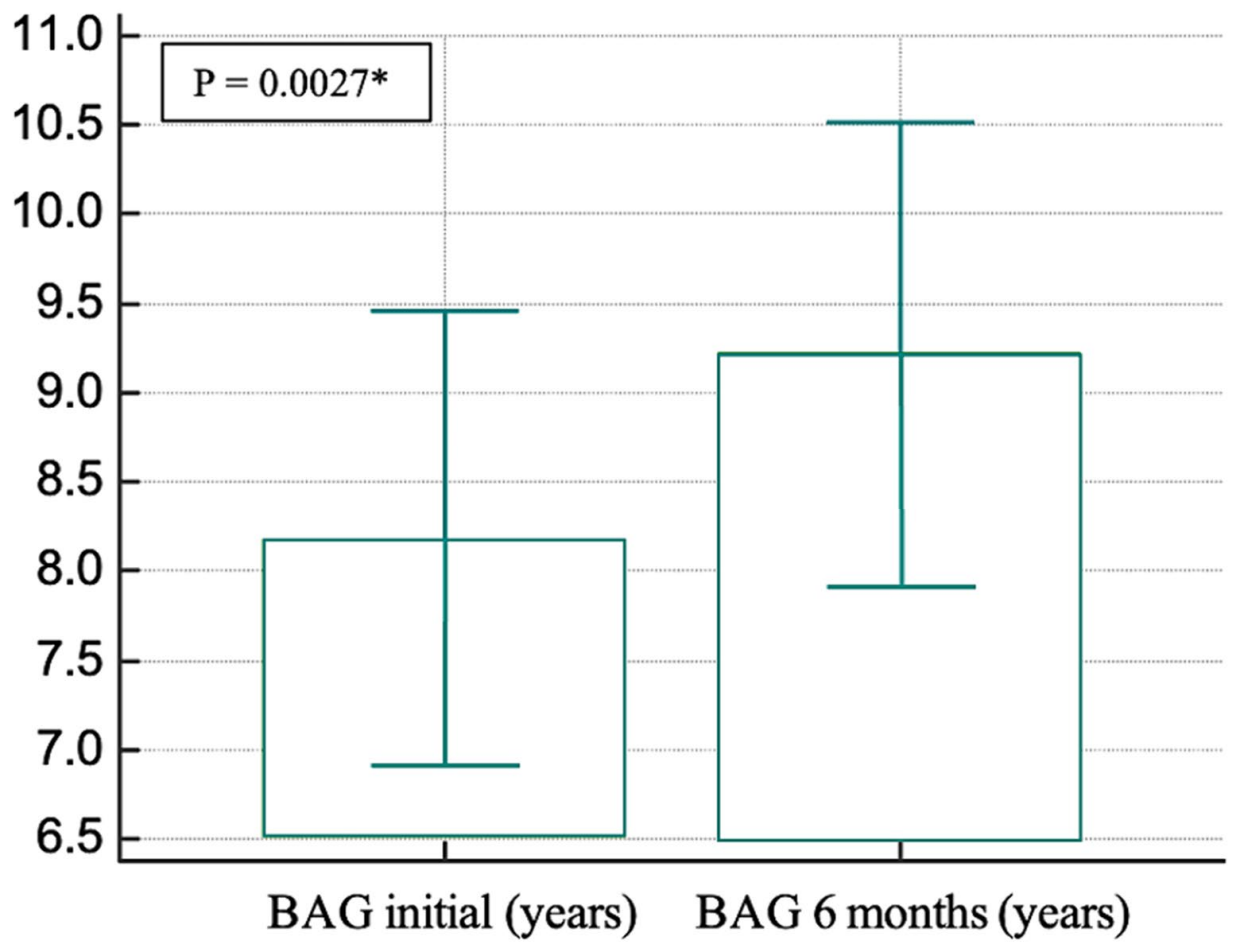
Bar graphs showing the results of the paired *t*-test in which we compared BAG by initial and 6-month status. BAG, Brain age gap.

In the longitudinal analysis involving all 123 subjects with no missing data at either time point, a paired *t*-test revealed a significant group difference (mean difference = 1.0363 years, *t* = 3.066, *p* < 0.05) between the initial and 6-month BAG (Figure 2).

**Figure 3:**
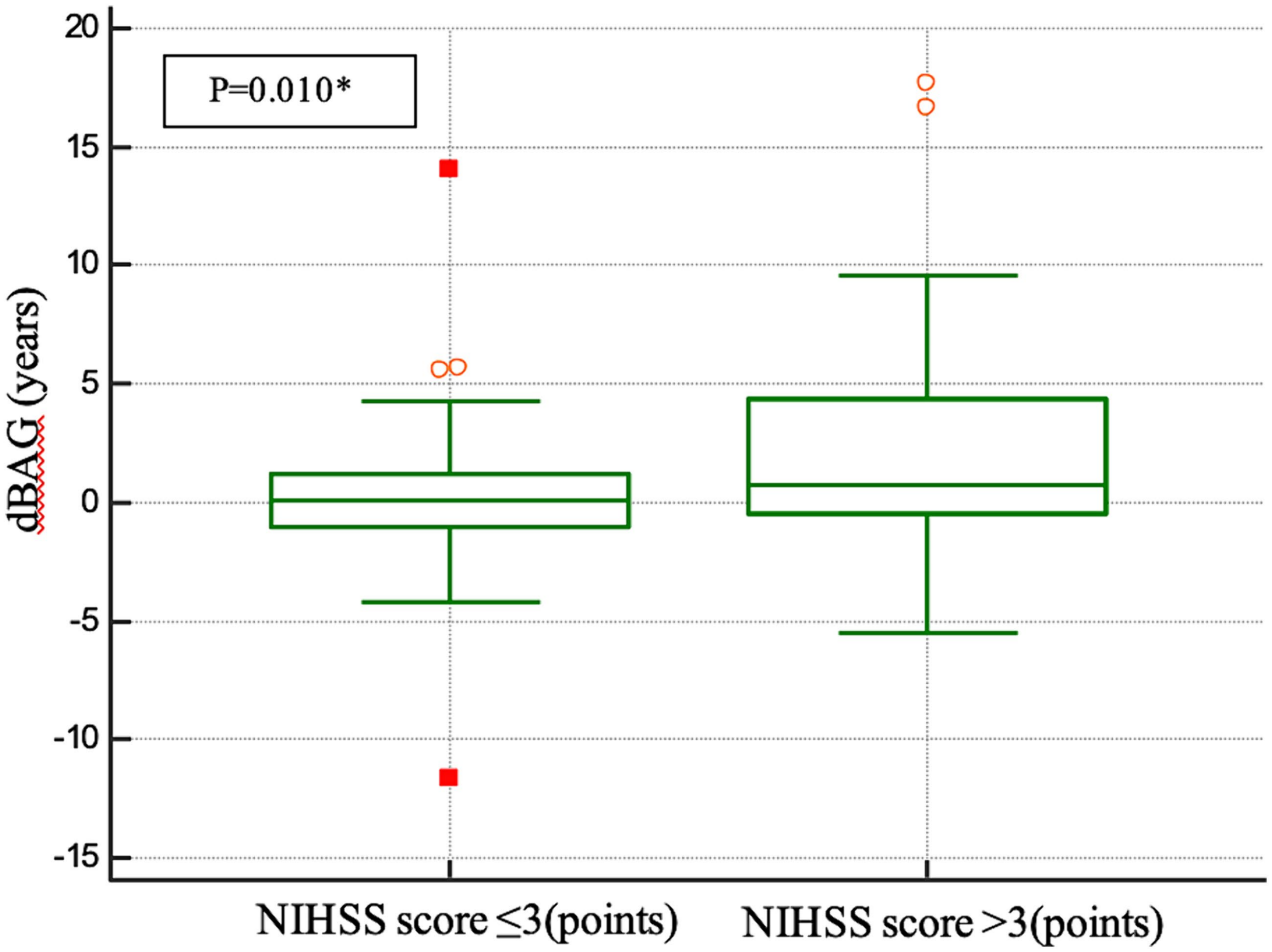
Box-and-whisker graph showing the results of the independent-samples *t*-test, in which we compared the dBAG by dichotomizing the outcome groups according to the median NIHSS score at admission. dBAG, difference between initial and 6-month BAG.

In terms of acute stroke severity, the results revealed that the dBAG was significantly greater in the higher-NIHSS group (dBAG = 1.922 ± 4.14) than in the lower-NIHSS group (dBAG = 0.192 ± 3.13) after adjustment for confounders, including chronological age, the square of chronological age, sex, and TIV (*F*_1,117_ = 7.339, *p* = 0.008, *η*^2^ = 0.059; Figure 3).

### Poststroke outcome factors and brain aging

The statistical results of the associations between PBAD at 6 months and poststroke outcome factors are summarized in Table 3. In terms of stroke-in-evolution and poststroke mRS scores, there were no significant differences in PBAD at 6 months among the study groups (stroke-in-evolution: *F*_1,119_ = 0.747, *p* = 0.389, *η*^2^ = 0.006; poststroke mRS score: *F*_1,119_ = 2.330, *p* = 0.130, *η*^2^ = 0.019). Moreover, the results of the Barthel Index score revealed a significant difference in PBAD at 6 months among the three study groups (*F*_2,118_ = 4.637, *p* = 0.012, *η*^2^ = 0.073). According to the *post-hoc* analyses, participants with high Barthel Index scores had greater PBADs (PBAD = 0.199 ± 0.161) than those with medium Barthel Index scores (PBAD = 0.117 ± 0.123, *p* = 0.004). However, there was no significant difference from the lower Barthel Index score group (PBAD = 0.141 ± 0.104, *p* = 0.054).

**Table 3:** Associations of the estimated brain age gap for the 6-month follow-up proportional brain age difference (PBAD 6 months) with outcome factors (*n* = 123).

Variables	Total		PBAD, 6 months	*p*-value
	*n*	%	Mean ± SD	
Stroke in evolution				0.389
Yes	10	8.13%	0.117 ± 0.109	
No	113	91.87%	0.158 ± 0.139	
Barthel index score				0.012*
≤60	39	31.71%	0.141 ± 0.104	
>60, ≤90	39	31.71%	0.117 ± 0.123	
>90	45	36.59%	0.199 ± 0.161	
Poststroke mRS score				0.130
≤3	70	56.91%	0.172 ± 0.149	
>3	53	43.09%	0.132 ± 0.116	

The data are presented as the means ± standard deviations.

## Discussion

The results of this study suggest that stroke accelerates the trajectory of brain aging trajectory, even in patients with small-volume infarcts. Our analyses revealed a significant group difference between the initial and 6-month BAG, suggesting that there is accelerated brain aging over a 6-month period after a stroke, with a mean difference of 1.04 years. According to previous studies, ischemic stroke leads to both acute and long-term inflammatory responses (DeLong et al., 2022). Chronic inflammation aggravates existing diseases as well as influences factors associated with aging (Germolec et al., 2018), which may explain the accelerated brain aging process poststroke. Furthermore, neuroimaging markers of brain aging, such as total brain volume and increased white matter hyperintensity load, have been linked to both vascular brain injury prior to a stroke event and continued brain atrophy and neurodegeneration, which may also shed light on the possible mechanism of accelerated brain aging process poststroke (Egorova et al., 2019). Moreover, small subcortical infarcts are the result of occlusions of small perforating brain arteries, also referred to as atherosclerosis (Nah et al., 2010). This typically causes hardening of the arterial wall and can lead to progressive stenosis and hypoperfusion (Xu et al., 2017). All these mechanisms are associated with local ischemia, which leads to neurodegeneration and progressive cognitive decline (Blinkouskaya et al., 2021). Cortical thinning may also be an imaging signature of vascular cognitive impairment. One study indicated that cortical thinning 3 years poststroke is greater in cognitively impaired survivors and that normal age-driven cortical thickness asymmetry loss is amplified in stroke patients (Salah Khlif et al., 2022).

In particular, the predominant stroke subtype among the patients included in this study was small-vessel occlusion, suggesting that our findings are particularly relevant to this etiology. Cerebral small-vessel disease (CSVD) can lead to several different clinical manifestations, including ischemic lacunar stroke, intracerebral hemorrhage, and vascular dementia (Singh et al., 2023). Age-related pathologies in small cerebral vessels, including arteriosclerosis, venule collagenosis, amyloid accumulation, and lipohyalinosis, are commonly found in aged brains (Brown and Thore, 2011; Farkas and Luiten, 2001). Current evidence suggests that even before clinical events, SVD lesions are already present, causing accelerated age-related cognitive and physical functional decline with diffuse structural and functional brain abnormalities (Chung et al., 2023). Another study has shown that SVD is very prevalent in young stroke patients and that cerebral aging seems to be accelerated by 10–20 years in these patients, which may indicate increased vulnerability to vascular risk factors (Arntz et al., 2016).

In particular, we found that for patients under the chronological age of 50 years, less accelerated brain aging after stroke was observed. As shown in Figure 1, the stroke participants’ ages in our study were above the normal trend (orange circles and green crosses above the black line) at both the initial and 6-month follow-ups. However, the brown dotted line tends to cross the blue line at points above 50 years of chronological age. This means that, in this longitudinal analysis, stroke patients above 50 years of chronological age exhibited more significant brain aging than patients under 50 years of age (lower chronological quartiles), indicating that younger patients may have greater physiological compensation ability and neuroplasticity over stroke episodes in the long term. A stroke may cause more pronounced impairment to the brain in chronologically older patients. A previous study revealed that aging is the strongest non-modifiable risk factor for ischemic stroke. Additionally, older stroke patients have higher mortality and morbidity rates and generally have poorer functional recovery than their younger counterparts (Roy-O’Reilly and McCullough, 2018). A previous prospective and community-based study reported that age independently influences stroke outcome selectively in ADL-related aspects (BIs) but not in neurological aspects, suggesting a poorer compensatory ability in elderly stroke patients (Nakayama et al., 1994).

Furthermore, we observed a significant difference between the group with a higher NIHSS score, defined as a score >3 points at admission, and the group with an elevated dBAG. This suggests that in small-volume infarction patients with more severe stroke, brain aging after stroke accelerates more rapidly over a 6-month period than in those with minor stroke severity. A recent study demonstrated that a higher relative brain age and a higher baseline NIHSS score were independently associated with worse poststroke outcomes (Bretzner et al., 2022). Another study revealed no significant association between lesion volume and NIHSS score at baseline with age prediction at 3 months (Egorova et al., 2019). Our study is the first to identify a possible correlation of a long-term difference in brain age gap with the NIHSS score at baseline, indicating that there is accelerated brain aging after small-volume infarctions, especially in patients with greater stroke severity.

In previous studies, brain age prediction in stroke patients was highly reliable but had limited sensitivity to cognitive performance and response to cognitive training (Richard et al., 2020). Although this study does not provide strong support for the utility of poststroke outcome factors as sensitive measurements for the aging process of the brain, our results suggest that the Barthel Index score might provide more information as a measure of cognitive or brain reserve. Future research is needed to validate the presumed relationships between poststroke outcome factors over an extended follow-up period.

This study has several limitations that warrant careful consideration. First, our study encompasses a relatively small dataset of stroke patients (*n* = 123), suggesting the potential requirement for additional complementary clinical investigations to increase the statistical robustness. Second, the absence of a control group poses limitations in discerning the potential correlations between vascular risk factors and brain aging. Subsequent investigations should consider the inclusion of a matched control group that is matched in terms of chronological age to facilitate comprehensive evaluations in this regard.

In conclusion, stroke accelerates the process of brain aging, even in patients with small-volume infarcts. This phenomenon is particularly impactful in patients aged 50 years and above and with greater stroke severity at admission, and poststroke functional outcomes are closely linked to accelerated brain aging. Further studies are needed to explore the mechanisms underlying the accelerated brain aging observed in stroke patients, with a particular focus on the structural alterations and neuroplasticity of the brain following minor strokes.

## Data Availability

The raw data supporting the conclusions of this article will be made available by the authors, without undue reservation.

## Footnotes

^1^ www.volbrain.upv.es

^2^ http://fsl.fmrib.ox.ac.uk/fsl/fslwiki/
